# Delta-radiomics signature predicts treatment outcomes after preoperative chemoradiotherapy and surgery in rectal cancer

**DOI:** 10.1186/s13014-019-1246-8

**Published:** 2019-03-12

**Authors:** Seung Hyuck Jeon, Changhoon Song, Eui Kyu Chie, Bohyoung Kim, Young Hoon Kim, Won Chang, Yoon Jin Lee, Joo-Hyun Chung, Jin Beom Chung, Keun-Wook Lee, Sung-Bum Kang, Jae-Sung Kim

**Affiliations:** 1 Department of Radiation Oncology, Seoul National University College of Medicine, 101 Daehak-ro, Jongno-gu, Seoul, 03080 Republic of Korea; 2 Department of Radiation Oncology, Seoul National University College of Medicine, Seoul National University Bundang Hospital, 82 Gumi-ro 173beon-gil, Bundang-gu, Seongnam, 13620 Republic of Korea; 3 Division of Biomedical Engineering, Hankuk University of Foreign Studies, 81 Oedae-ro, Mohyeon-eup, Cheoin-gu, Yongin, 17035 Republic of Korea; 4 Department of Radiology, Seoul National University College of Medicine, Seoul National University Bundang Hospital, 82 Gumi-ro 173beon-gil, Bundang-gu, Seongnam, 13620 Republic of Korea; 5 Department of Internal Medicine, Seoul National University College of Medicine, Seoul National University Bundang Hospital, 82 Gumi-ro 173beon-gil, Bundang-gu, Seongnam, 13620 Republic of Korea; 6 Department of Surgery, Seoul National University College of Medicine, Seoul National University Bundang Hospital, 82 Gumi-ro 173beon-gil, Bundang-gu, Seongnam, 13620 Republic of Korea

**Keywords:** Rectal cancer, Radiomics, Delta-radiomics, Chemoradiotherapy

## Abstract

**Background:**

To develop and compare delta-radiomics signatures from 2- (2D) and 3-dimensional (3D) features that predict treatment outcomes following preoperative chemoradiotherapy (CCRT) and surgery for locally advanced rectal cancer.

**Methods:**

In total, 101 patients (training cohort, *n* = 67; validation cohort, *n* = 34) with locally advanced rectal adenocarcinoma between 2008 and 2015 were included. We extracted 55 features from T2-weighted magnetic resonance imaging (MRI) scans. Delta-radiomics feature was defined as the difference in radiomics feature before and after CCRT. Signatures were developed to predict local recurrence (LR), distant metastasis (DM), and disease-free survival (DFS) from 2D and 3D features. The least absolute shrinkage and selection operator regression was used to select features and build signatures. The delta-radiomics signatures and clinical factors were integrated into Cox regression analysis to determine if the signatures were independent prognostic factors.

**Results:**

The radiomics signatures for LR, DM, and DFS were developed and validated using both 2D and 3D features. Outcomes were significantly different in the low- and high-risk patients dichotomized by optimal cutoff in both the training and validation cohorts. In multivariate analysis, the signatures were independent prognostic factors even when considering the clinical parameters. There were no significant differences in C-index from 2D vs. 3D signatures.

**Conclusions:**

This is the first study to develop delta-radiomics signatures for rectal cancer. The signatures successfully predicted the outcomes and were independent prognostic factors. External validation is warranted to ensure their performance.

**Electronic supplementary material:**

The online version of this article (10.1186/s13014-019-1246-8) contains supplementary material, which is available to authorized users.

## Background

After the landmark randomized trial [1], preoperative chemoradiotherapy (CCRT) followed by total mesorectal excision (TME) has been a standard treatment strategy for locoregionally advanced rectal cancer. However, efforts have been continuously made to promote risk-adaptive therapy. One such approach is local excision [2, 3] or even observation [4, 5], rather than TME, in good responders following CCRT to reduce the risk of impaired quality of life. Alternatively, adding chemotherapeutic agents or intensifying radiation doses may be attempted in patients with poor response or prognosis [6–8]. These strategies can be implemented with the help of treatment outcome predictors; however, there are still no tools explicitly available for this purpose.

Radiomics provide image features associated with clinical characteristics or outcomes that are extracted from medical images. Numerous studies have been conducted on various cancer types, including lung cancer [9], glioma [10], and head and neck cancer [11] as well as proposed radiomics-based predictors of good performance. Radiomics models for rectal cancer have recently been developed using computed tomography (CT), magnetic resonance imaging (MRI), and positron emission tomography (PET) [12–18]. Some researchers have built radiomics models to predict pathologic response to CCRT [12, 13, 17, 18]. Meng et al. [14] reported a radiomics signature to predict disease-free survival (DFS) using pretreatment MRI in which the models could predict the treatment response or prognosis with acceptable predictability. Nonetheless, clinical response to treatment is another important indicator and may improve the performance of the models. Although treatment response is an important prognostic factor, it cannot describe the entire details of response. In this context, some researchers have examined delta-radiomic features, which are the differences in radiomic features before and after treatment. Delta-radiomics deals with serial changes in images, which is one of the major parts of radiologic studies. Delta-radiomics features have been reported to be associated with treatment response or outcome [19, 20]; however, reports on delta-radiomics in rectal cancer are less [21].

In this paper, we have focused on applying delta-radiomics features extracted from T2-weighted MRI to build prediction signatures for treatment outcomes and compared the performances of 2- (2D) and 3-dimensional (3D) features.

## Methods

### Study population

This retrospective study was approved by the institutional review board of our hospital; the requirement of informed consent was waived. The protocol was compliant with the Health Insurance Portability and Accountability Act. We retrospectively enrolled patients with locally advanced (cT3–4 and/or cN1–2) biopsy-proven rectal adenocarcinoma treated at our institution with preoperative CCRT and TME between 2008 and 2015. Patients with the following were excluded: distant metastasis (DM) at the time of diagnosis, MRI with poor quality (e.g., artifact), or slice spacing of MRI not 4 mm (to minimize the influence of different voxel sizes). Included patients were randomly allocated to the training or validation cohort in a 2:1 ratio.

### Treatment outcome

We examined local recurrence (LR), DM, and DFS. LR and DM were defined as recurrences inside and outside the true pelvis, respectively. DFS was calculated as time from beginning of preoperative CCRT to death from any cause or recurrence.

### Image protocol

An MRI scan was obtained for each patient before preoperative CCRT (MRI-before) and before TME after completion of CCRT (MRI-after). MRI-after was acquired at 72 days (median; interquartile range, 70–78) after the start of CCRT. MRI was performed using 1 .5T Gyroscan Intera, 3 T Achieva, or 3 T Ingenia MR scanners (Philips Medical Systems, Best, Netherlands). The protocol included T2-weighted sequences using the following parameters: repetition time, 2424–8296 ms; echo time, 92–120 ms; flip angle, 90°; slice thickness, 3 mm; slice spacing, 4 mm; matrix, 512 × 512–576 × 576.

### Segmentation

**Fig. 1:**
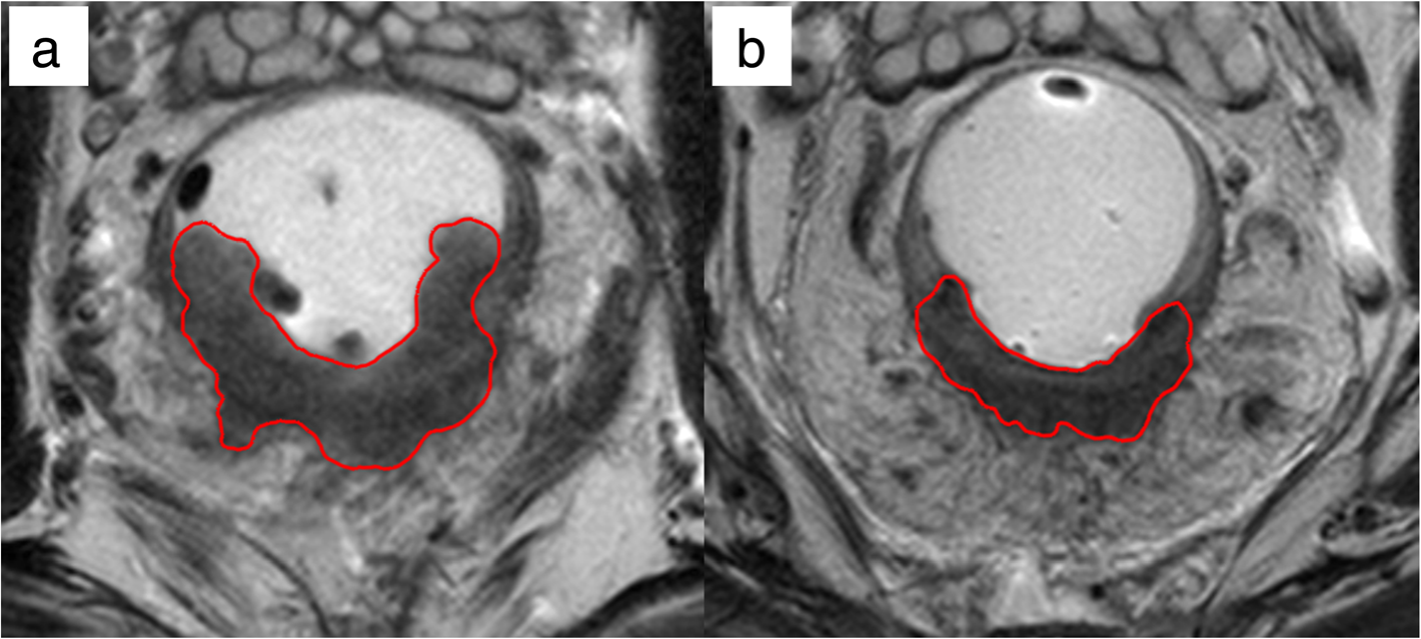
Examples of tumor segmentation on MRI acquired (**a**) before and (**b**) after preoperative CCRT

Each region of interest (ROI) was segmented on all T2-weighted axial with reference to diffusion-weighted imaging (DWI) sequences. On MRI-before, the ROI was delineated on the tumor with an area of low to intermediate signal intensity on T2-weighted images, excluding the intestinal lumen. The ROI on MRI-after was defined as residual tumor and/or rectal tissues with abnormal signal intensity on T2-weighted images where tumor preexisted [13]. Bladder urine of approximately 1-cm^3^ sphere volume was drawn to obtain average pixel value of bladder urine which was used for normalization. Segmentation of all patients was performed manually using the Eclipse system (Varian Medical Systems, Palo Alto, CA, USA) by a radiation oncologist with 12-year experience in gastro-intestinal tumor. Representative examples of tumor segmentations are demonstrated on Fig. 1.

### Image preprocessing and feature extraction

Image preprocessing and feature extraction were performed using in-house MATLAB R2017b software (MathWorks, Natick, MA, USA). For preprocessing, Collewet normalization algorithm [22] was used to reduce the differences between image acquisition protocols. All pixel values were normalized to average intensity of bladder urine [23] to improve image reproducibility. Pixel values were quantized into 64 levels with bladder urine signal intensity corresponding to the highest level. The 3-dimensional ROIs were isotropically resampled to 1 × 1 × 1-mm^3^ voxels. For 2-dimensional analysis, the ROI on the axial slice with the largest area was selected and resampled to 1 × 1-mm^2^ pixels.

Within each ROI, (a) volume (or area in 2-dimensional analysis), (b) 8 first-order features, (c) 15 texture features from gray level co-occurrence matrix, (d) 13 texture features from gray level run length matrix, (e) 13 texture features from gray level size zone matrix, and (f) 5 texture features from neighbor gray tone difference matrix were extracted. The details and list of the extracted features are described in Additional file 1: Appendix A and Table B1, respectively. The delta-radiomics feature was defined as the difference between features on MRI-before and MRI-after and calculated as follows:

$$ \mathrm{Delta}-\mathrm{radiomic}\ \mathrm{Feature}={\mathrm{Feature}}_{\mathrm{MRI}-\mathrm{after}}-{\mathrm{Feature}}_{\mathrm{MRI}-\mathrm{before}} $$

### Feature selection and statistical analysis

Robustness of each feature was evaluated by generating translated ROIs and calculating their features. The method was modified from the stability test introduced by Bologna et al. [24] Eight translated ROIs representing inter-observer variability were generated by translating ROI by ±1 mm in lateral and/or ± 1 mm in vertical directions; 0 mm in both directions yields the original ROI and is thus excluded from the robustness test. After extracting the features from the original ROI and 8 translated ROIs, intraclass correlation coefficient (ICC) values were calculated for each feature. Features with ICC > 0.9 in both 3D and 2D extraction in MRI-before and MRI-after were considered robust and selected. This process substituted the comparison of features derived from multiple observers.

The least absolute shrinkage and selection operator (LASSO) method was used to select core features and to develop score-based signatures in the training cohort. The final value of λ, a tuning parameter, was determined by 10-fold cross-validation, which gave minimum cross-validation error. A radiomics score (Rad score) was generated by linearly combining the selected core features and their respective coefficients. Consequently, the optimal cutoff of Rad score, making the greatest difference in outcome between the two groups divided by the cutoff, was determined.

The differences in clinical and treatment parameters between the training and validation cohort were evaluated using the Student’s *t*-test or chi-squared test, as appropriate. Survival outcomes were compared between these cohorts using the log-rank test. Univariate and multivariate analyses of clinical factors and radiomics scores were performed using the Cox proportional hazards model. Performance of the models were evaluated with area under the ROC curve (AUC) and Hosmer-Lemeshow goodness-of-fit test, and the relationship between radiomics scores was quantified using Pearson correlation coefficient and variance inflation factor (VIF). Variables with a significant association were integrated into multivariate analysis; the association was considered significant when *p* < 0.05. R software version 3.5.0 was used to perform all statistical analyses (http://www.r-project.org).

## Results

### Patients and treatment characteristics

A total of 101 patients were included in the analysis, with 67 in the training and 34 in the validation cohort. The median follow-up duration was 49.7 months (range, 9.3–99.4). Clinical characteristics of the two cohorts are summarized in Table 1. There was no significant difference between the two cohorts.

**Table 1:** Patient characteristics of training and validation cohort

		Training cohort (*N* = 67)	Validation cohort (*N* = 34)	*P*-value
Age		59.5 ± 11.6	62.5 ± 11.4	0.22^a^
Sex	Male	54 (80.6%)	22 (64.7%)	0.13^b^
	Female	13 (19.4%)	12 (35.3%)	
Clinical T stage	cT1–3	59 (88.1%)	30 (88.2%)	1.00^b^
	cT4	8 (11.9%)	4 (11.8%)	
Clinical N stage	cN0	9 (13.4%)	5 (14.7%)	1.00^b^
	cN1–2	58 (86.6%)	29 (85.3%)	
Dworak TRG	1	15 (22.4%)	4 (11.8%)	0.64^b^
	2	27 (40.3%)	16 (47.1%)	
	3	14 (20.9%)	8 (23.5%)	
	4	11 (16.4%)	6 (17.6%)	
Pathologic T stage	ypT0–2	37 (55.2%)	16 (47.1%)	0.57^b^
	ypT3–4	30 (44.8%)	18 (52.9%)	
Pathologic N stage	ypN0	46 (68.7%)	22 (64.7%)	0.86^b^
	ypN1–2	21 (31.3%)	12 (35.3%)	
Initial CEA	≤5 ng/mL	42 (62.7%)	24 (70.6%)	0.57^b^
	> 5 ng/mL	25 (37.3%)	10 (29.4%)	
Local recurrence	Yes	8 (11.9%)	2 (5.9%)	0.54^b^
	No	59 (88.1%)	32 (94.1%)	
Distant metastasis	Yes	16 (23.9%)	6 (17.6%)	0.64^b^
	No	51 (76.1%)	28 (82.4%)	

^a^Student’s t-test

_b_Chi-squared test

Abbreviations: *TRG* tumor regression grade, *CEA* carcinoembryonic antigen

All patients received radiotherapy doses of 50.4 Gy in 28 fractions to the primary tumor and regional lymphatics with the 2D (*n* = 18) or 3D (*n* = 83) technique. Additionally, 5-fluorouracil (*n* = 19) or capecitabine (*n* = 82) was administered concurrently with radiotherapy. TME-based surgery was performed at a median 48 days (range, 28–90) after the end of CCRT. Adjuvant chemotherapy was administered in 91 patients (90.1%); fluorouracil and leucovorin in 20, capecitabine in 36, uracil and tegafur in 7, and FOLFOX in 28 patients, respectively.

### Development and validation of Delta-Radiomics signature

The delta-radiomics signature was developed using the remaining 22 features after the robustness test. The 22 robust features with ICC > 0.9 are listed in Additional file 1: Appendix Table B2. LASSO Cox regression analysis was conducted in the training cohort to select radiomics features with non-zero coefficients. Rad scores predicting LR, DM, and DFS are as follows:

$$ \mathrm{LR}\ 3\mathrm{D}\ \mathrm{Radscore}=-5.9627417\times {10}^{-5}\ \mathrm{x}\ \mathrm{Volume}+4.0761146\times {\mathrm{Int}}_{\mathrm{Energy}}-135.5705805\times {\mathrm{GLCM}}_{\mathrm{Energy}}+286.7201809\times {\mathrm{GLCM}}_{\mathrm{SumAverage}}+2.7222298\ \mathrm{x}\ {10}^{-3}\times {\mathrm{GLCM}}_{\mathrm{Autocorrelation}}+0.1212618\times {\mathrm{GLSZM}}_{\mathrm{LZLGLE}}-263.8275908\times {\mathrm{NGTDM}}_{\mathrm{Coarseness}} $$

$$ \mathrm{LR}\ 2\mathrm{D}\ \mathrm{Radscore}=-3.9078995\times {10}^{-4}\times \mathrm{Volume}+2.4888091\times {\mathrm{Int}}_{\mathrm{Energy}}-22.2879655\times {\mathrm{GLCM}}_{\mathrm{Energy}}+432.3870771\times {\mathrm{GLCM}}_{\mathrm{SumAverage}}-8.7912561\times {\mathrm{GLRLM}}_{\mathrm{SRLGLE}}-37.6853716\times {\mathrm{NGTDM}}_{\mathrm{Coarseness}} $$

$$ \mathrm{DM}\ 3\mathrm{D}\ \mathrm{Radscore}=2.0001257\times {\mathrm{Int}}_{\mathrm{Energy}}+9.0766595\times {10}^{-5}\times {\mathrm{GLCM}}_{\mathrm{SumVariance}}-36.4133193\times {\mathrm{GLRLM}}_{\mathrm{LRLGLE}}+1.3710706\ \mathrm{x}\ {10}^{-4}\times {\mathrm{GLRLM}}_{\mathrm{LRHGLE}}+2.0565009\times {10}^{-8}\times {\mathrm{GLSZM}}_{\mathrm{LZHGLE}}-108.1326981\times {\mathrm{NGTDM}}_{\mathrm{Coarseness}} $$

$$ \mathrm{DM}\ 2\mathrm{D}\ \mathrm{Radscore}=0.4580866\times {\mathrm{Int}}_{\mathrm{Energy}}-45.2277485\times {\mathrm{NGTDM}}_{\mathrm{Coarseness}} $$

$$ \mathrm{DFS}\ 3\mathrm{D}\ \mathrm{Radscore}=1.6123539\times {\mathrm{Int}}_{\mathrm{Energy}}+7.9686750\times {10}^{-5}\times {\mathrm{GLCM}}_{\mathrm{SumVariance}}-32.6172005\times {\mathrm{GLRLM}}_{\mathrm{LRLGLE}}+1.1112033\ \mathrm{x}\ {10}^{-4}\times {\mathrm{GLRLM}}_{\mathrm{LRHGLE}}-101.5991090\times {\mathrm{NGTDM}}_{\mathrm{Coarseness}} $$

$$ \mathrm{DFS}\ 2\mathrm{D}\ \mathrm{Radscore}=0.3188674\times {\mathrm{Int}}_{\mathrm{Energy}}-44.9766973\times {\mathrm{NGTDM}}_{\mathrm{Coarseness}} $$

**Fig. 2:**
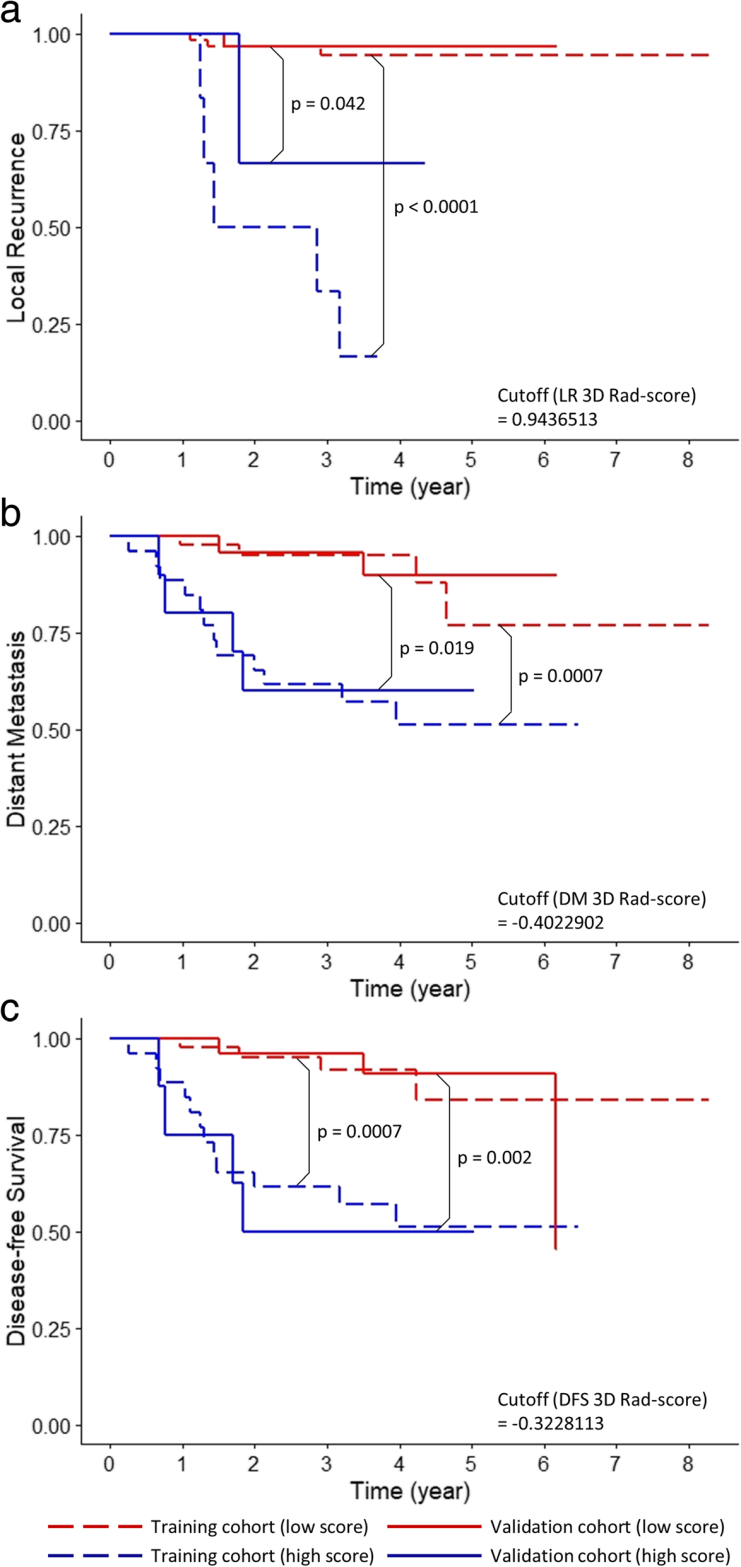
Kaplan–Meier curves of (**a**) local recurrence, (**b**) distant metastasis, and (**c**) disease-free survival according to optimal cutoffs of 3D Rad scores. P-values from log-rank test are shown

**Fig. 3:**
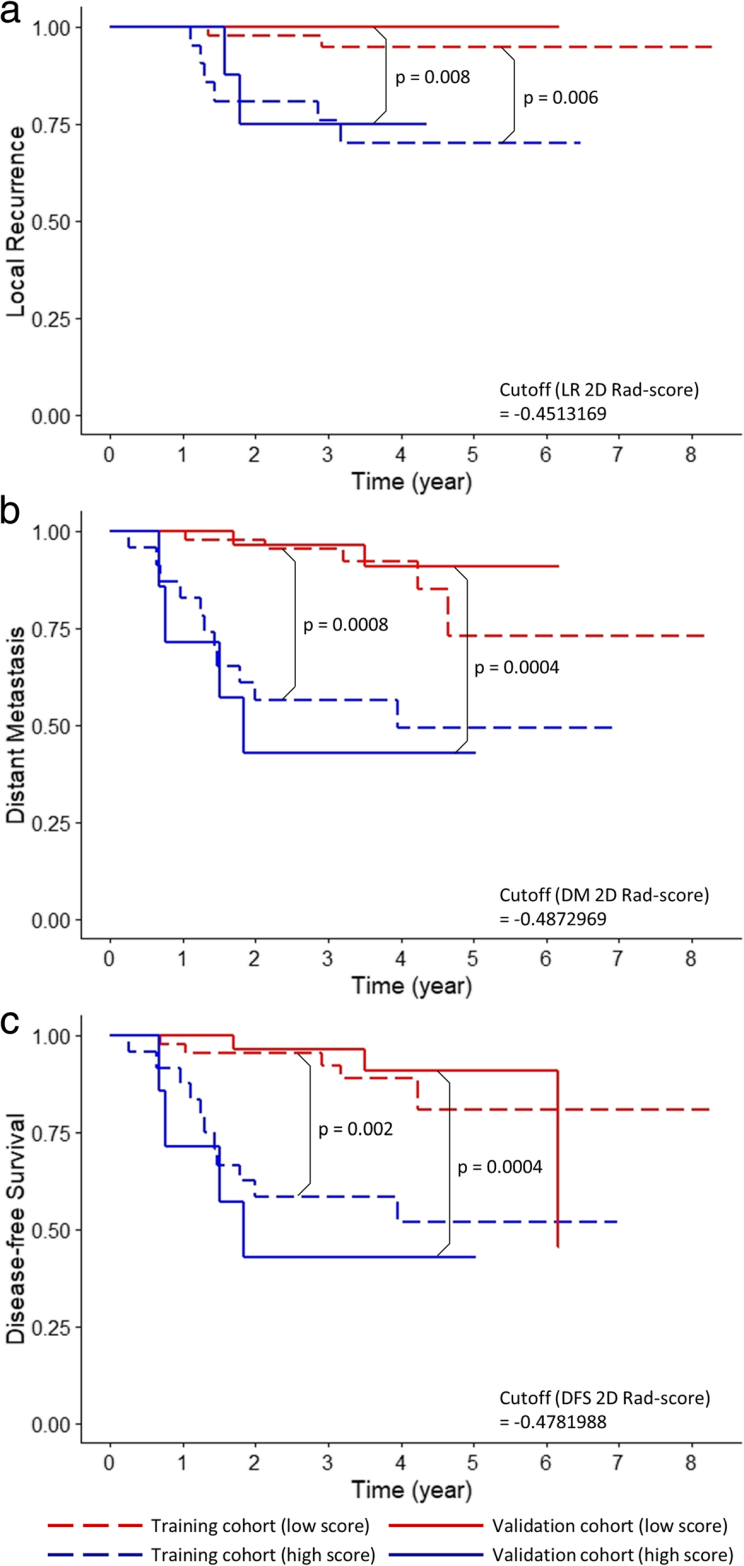
Kaplan–Meier curves of (**a**) local recurrence, (**b**) distant metastasis, and (**c**) disease-free survival according to optimal cutoffs of 2D Rad scores. *P*-values from log-rank test are shown

Optimal cutoff values of Rad scores were determined and used to divide the cohort into high- and low-risk groups, and a higher score was correlated to a higher risk. In the training cohort, all Rad scores were significantly associated with respective outcomes (all *p* < 0.05, log-rank test). The prognostic performance of all Rad scores was validated in a randomly selected cohort. All Rad scores significantly stratified the risk in the 2 groups (all p < 0.05, log-rank test). The Kaplan-Meier survival curves for LR, DM, and DFS according to 3D and 2D Radscores are demonstrated on Fig. 2 and Fig.3, respectively. The AUC and Hosmer-Lemeshow Chi-square values suggest that the predictability of the signatures is acceptable and are detailed on Additional file 1: Appendix Table C.

### Integration with clinical features

Univariate analysis followed by multivariate analysis was performed to verify radiomics scores as independent prognostic factors for the respective endpoints in the combined cohort. Detailed results of the analyses are presented in Additional file 1: Appendix Table D1–3. All radiomics scores, regardless of 2D or 3D signature, were significantly associated with the corresponding outcomes on multivariate analysis.

### Comparisons of 3D and 2D signatures

**Fig. 4:**
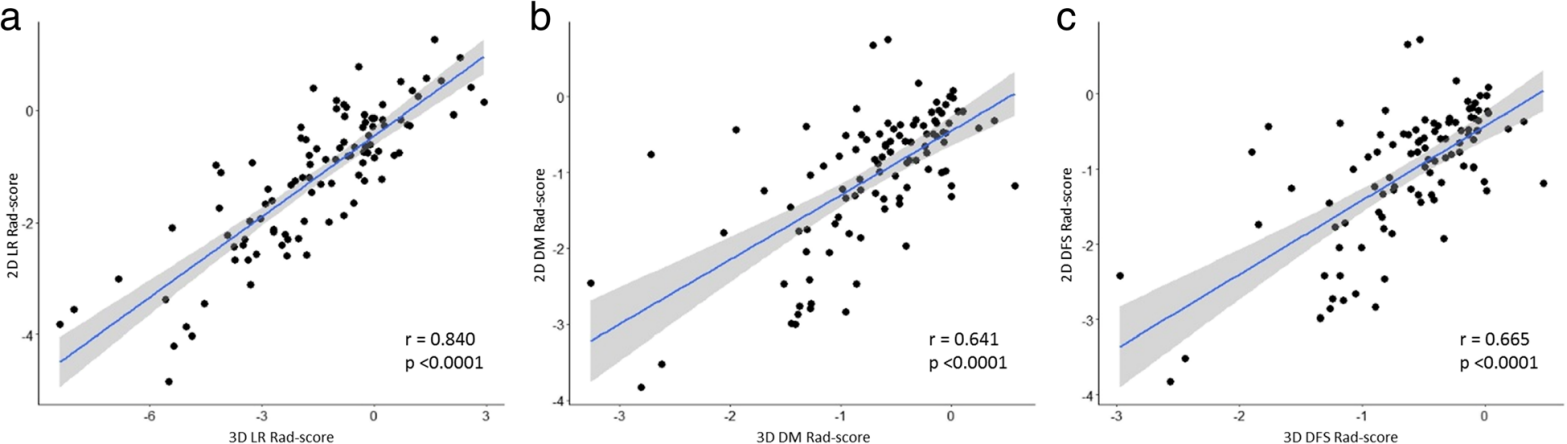
The scatterplots between 3D and 2D Rad scores of the entire cohort (*n* = 101) predicting (**a**) local recurrence, (**b**) distant metastasis, and (**c**) disease-free survival. Linear fit lines and 95% confidence intervals were drawn, and presented coefficients and p-values were calculated using Pearson correlation coefficient

Correlations between 3D and 2D Rad scores were investigated in the 101 patients. Pearson correlation coefficients of 3D and 2D Rad scores for LR, DM, and DFS were 0.840 (95% CI = 0.771–0.889, *p* < 0.0001, VIF = 3.39), 0.641 (95% CI = 0.510–0.743, p < 0.0001, VIF = 1.70), and 0.665 (95% CI = 0.540–0.761, p < 0.0001, VIF = 1.79), respectively. The scatterplot of Rad scores and their correlations are shown in Fig. 4.

The value of C-index was calculated and compared for each Rad score. In terms of all outcomes, the C-indices between the 2D and 3D Rad scores, both as continuous and binary variables divided by cutoff, were not significant (Table 2).

**Table 2:** Comparison of C-indices of 3D and 2D Rad-scores. The 95% confidence interval for each C-index is presented

	C-index		*P*-value
	3D	2D	
LR Rad-score			
Continuous	0.893 ± 0.035	0.873 ± 0.050	0.24
Binary	0.951 ± 0.026	0.926 ± 0.054	0.29
DM Rad-score			
Continuous	0.783 ± 0.039	0.774 ± 0.042	0.38
Binary	0.894 ± 0.048	0.911 ± 0.040	0.64
DFS Rad-score			
Continuous	0.788 ± 0.039	0.763 ± 0.041	0.22
Binary	0.897 ± 0.046	0.886 ± 0.048	0.41

Abbreviations: *LR* local recurrence, *DM* distant metastasis, *DFS* disease-free survival

## Discussion

In the present study, we developed and validated the prognostic role of delta-radiomics signatures in locally advanced rectal cancer treated with preoperative CCRT and surgery. Furthermore, we compared the performance of 2D and 3D radiomics features. To our knowledge, this is the first publication to incorporate delta-radiomics features to predict recurrences in rectal cancer.

A good response to preoperative CCRT is consistently associated with improved treatment outcomes of rectal cancer [25, 26]. Although pretreatment radiomics features predict pathologic tumor response, they do not contain all of the information regarding response. Images following preoperative CCRT can reveal indicators of tumor response. Tumor regression grade according to post-treatment T2-weighted MRI is correlated with pathologic tumor regression grade [27, 28]. Thus, we hypothesized that delta-radiomics features on T2-weighted MRI have prognostic power. A recent study showed correlation between delta-radiomics features and clinical response in rectal cancer [21]. Analyzing 16 patients, the study provided an evidence of clinical significance of delta-radiomics features. Since there are no studies with large patients concerning delta-radiomics, however, we strictly limited the number of features included in the investigation. We only included the features that are widely used in radiomics studies, and the image preprocessing step was onefold. Furthermore, we included the features with ICC > 0.9 in all 3D and 2D analyses, leaving 22 features for LASSO regression.

The developed Rad scores were successfully validated in a randomly selected cohort. Some radiomics features may be closely related to clinical factors, thus the signatures should be independent prognostic factors in multivariate analysis to be valuable. Rad scores along with clinical factors consistently reported to be prognostic were independently associated with treatment outcomes. Remarkably, post-treatment pathologic characteristics such as pathologic stage and tumor regression grade are incorporated in the analysis. The results suggest that delta-radiomics features may contain more information than microscopic findings, e.g., tumor genotype or microenvironment.

The developed signatures are believed to be useful in daily practice. There is no consensus regarding the use of adjuvant chemotherapy after preoperative CCRT and surgery. Subgroups that may benefit from adjuvant chemotherapy have been reported [29–31]. It is generally hypothesized that patients at high risk of recurrence, usually those with distant metastasis, benefit from adjuvant chemotherapy. Most of the patients (90.1%) in our study received adjuvant chemotherapy, suggesting that, for patients with low Rad scores, adjuvant chemotherapy can be omitted.

We compared 2D and 3D delta-radiomics signatures in predicting outcomes. One major advantage of the radiomics approach is that it can represent properties of the whole tumor. However, in the case of 2D features, only part of the tumor is segmented. Therefore, there are concerns regarding the power of 2D radiomics features. The main advantage of 2D radiomics features is the convenience in investigation and application; investigators or users only need to delineate the tumor on 1 representative slice. In addition, 2D features, particularly slice thickness and spacing, may be less dependent on the image-acquiring protocol. Several authors have utilized 2D radiomics features of rectal cancer and reported their predictive power [32–34]. As rectal cancer usually grows along the wall and has an irregular shape, the segmented whole tumor may not represent its actual shape [35]; hence, 2D radiomics features need to be further studied. Regarding all outcomes, both 2D and 3D Rad scores were independent prognostic factors on multivariate analysis. By comparing C-index, 2D and 3D Rad scores were not statistically different in prognostic power, possibly because of the high correlation between scores. Hence, our data suggest that 2D delta-radiomics features can be investigated as a good surrogate for 3D features of rectal cancer.

One of the drawbacks of our study is the exclusion of functional images such as DWI or other modalities such as CT and PET. Several studies have reported the correlation between DWI parameters and response or outcomes after CCRT [36–38]. Recent work by Giannini and colleagues demonstrated the role of PET-derived radiomics features in predicting treatment response [18]. We believe that the performance of delta-radiomics signature would improve with the incorporation of other sequences or modalities. We hope that radiomics features from various images can be used in subsequent delta-radiomics investigations. Another limitation of the study is the different parameters of the analyzed T2-weighted images. We normalized the pixel intensity using Collewet’s method and urine intensity and resampled the voxels or pixels into isometric cubes or squares. Nonetheless, the preprocessing steps cannot fully compensate for the differences. However, we believe that the radiomics signature should be applicable to various image protocols for widespread clinical use. In that context, the wide applicability of our signatures needs to be tested in MRIs from other institutions.

In conclusion, we developed radiomics scores to predict treatment outcomes after preoperative CCRT and surgery. The results support further investigation of delta-radiomics features in rectal cancer. The 2D and 3D delta-radiomics features were similarly informative. External validation of our signatures is necessary to ensure their performance.

## Additional file

Additional file 1: Appendix A. Extracted radiomics features. (DOCX 37 kb)

## Abbreviations

CCRT: Chemoradiotherapy
CI: Confidence interval
DFS: Disease-free survival
DM: Distant metastasis
DWI: Diffusion-weighted image
ICC: Intraclass correlation coefficient
LASSO: Least absolute shrinkage and selection operator
LR: Local recurrence
ROI: Region of interest
TME: Total mesorectal excision
