# Supplementary material for: Delta-radiomics signature predicts treatment outcomes after preoperative chemoradiotherapy and surgery in rectal cancer
Source: Radiat Oncol. 2019 Mar 12;14:43. doi: 10.1186/s13014-019-1246-8 (PMC6417065; doi:10.1186/s13014-019-1246-8)
Supplement: Supplementary file 1 — Appendix A. Extracted radiomics features. (DOCX 37 kb) [file 13014_2019_1246_MOESM1_ESM.docx]

**Appendix A**

**Extracted radiomics features**

**1. Geometry**

N_V_ and N_A_ are the number of isotropically resampled voxels (1 x 1 x 1 mm^3^) of whole tumor volume and pixels (1 x 1 mm^2^) of slice with the largest area, respectively.

1.1 Volume

$$volume=N_{V} ({mm}^{3})$$

1.2 Area

$$area=N_{A} ({mm}^{2})$$

**2. First-order features**

First order features describe the distribution of gray values within a region of interest. The number of voxels in given ROI is denoted with ***N***. The probability vector of the first order histogram with ***N_g_*** (=64) discrete bins is denoted with ***P***.

2.1. Mean

$$mean=u=\sum_{i=1}^{N_{g}} i \times P(i)$$

2.2. Variance

$$variance=\sigma^{2}=\sum_{i=1}^{N_{g}} {(i-u)}^{2}\times P(i)$$

2.3. Interquartile range

$$interquartile range=P_{75\%}-P_{25\%}$$

2.4. Energy

$$energy=\sum_{i=1}^{N_{g}} \left( N\times P\left( i \right) \right)^{2}$$

2.5. Entropy

$$entropy=-\sum_{i=1}^{N_{g}} \log_{2} P(i)\times P(i)$$

2.6. Uniformity

$$uniformity=\sum_{i=1}^{N_{g}} {P(i)}^{2}$$

2.7. Skewness

$$skewness=\frac{\sum_{i=1}^{N_{g}} {(i-u)}^{3}\times P(i)}{\sigma^{3}}$$

2.8. Kurtosis

$$kurtosis= \frac{\sum_{i=1}^{N_{g}} {(i-u)}^{4}\times P(i)}{\sigma^{4}}-3$$

**3. Gray-level co-occurrence matrix (GLCM) based texture features**

A GLCM of size N_g_ x N_g_ (=64 x 64) is denoted with ***P*** and describes textural information of given ROI. The **P**(*i*, *j*) is defined as the number of times a voxels of intensity *i* and the voxel in a distance δ of intensity j. The distance vector δ is from $\left\{ \left( x,y,z \right)|x\in\left( -1,0,1 \right) and y\in\left( -1,0,1 \right) and z\in\left( -1,0,1 \right) and (x,y,z)\neq(0,0,0) \right\}$. In case of 2D features, δ is from $\left\{ \left( x,y \right)|x\in\left( -1,0,1 \right) and y\in\left( -1,0,1 \right) and (x,y)\neq(0,0) \right\}$. To account for discretization length differences, neighbors at a distance of 1, $\sqrt{2}$, $\sqrt{3}$ were given a weight of 1, $1/\sqrt{2}$, $1/\sqrt{3}$, respectively.

• p(i,j) = ${P(i,j)}/{\sum P(i,j)}$

• μ = mean of p(i,j)

• μ_x_ = mean of p_x_(i)

• μ_y_ = mean of p_y_(j)

• σ = standard deviation of p(i,j)

• p_x+y_(k) = $\sum_{i}^{N_{g}} \sum_{j}^{N_{g}} p\left( i,j \right)$, $i+j=k$

• p_x-y_(k) = $\sum_{i}^{N_{g}} \sum_{j}^{N_{g}} p\left( i,j \right)$, $\left| i-j \right|=k$

3.1. Energy

$$energy=\sum_{i=1}^{N_{g}} \sum_{j=1}^{N_{g}} {p(i,j)}^{2}$$

3.2. Contrast

$$contrast=\sum_{i=1}^{N_{g}} \sum_{j=1}^{N_{g}} \left( i-j \right)^{2}p(i,j)$$

3.3. Entropy

$$entropy=-\sum_{i=1}^{N_{g}} \sum_{j=1}^{N_{g}} \log_{2} \left[ P\left( i,j \right) \right]\times p(i,j)$$

3.4. Homogeneity

$$homogeneity=\sum_{i=1}^{N_{g}} \sum_{j=1}^{N_{g}} \frac{p(i,j)}{1+\left| i-j \right|}$$

3.5. Correlation

$$correlation=\frac{1}{\sigma}\sum_{i=1}^{N_{g}} \sum_{j=1}^{N_{g}} (i-\mu)(j-\mu)p(i,j)$$

3.6. Variance

$$variance=\frac{1}{{2N_{g}}^{2}}\sum_{i=1}^{N_{g}} \sum_{j=1}^{N_{g}} \left[ \left( i-\mu\right)^{2}p\left( i,j \right)+\left( j-\mu\right)^{2}p(i,j) \right]$$

3.7. Sum Average

$$sum average=\frac{1}{{2N_{g}}^{2}}\sum_{i=1}^{N_{g}} \sum_{j=1}^{N_{g}} \left( i+j \right)p(i,j)$$

3.8. Dissimilarity

$$dissimilarity=\sum_{i=1}^{N_{g}} \sum_{j=1}^{N_{g}} \left| i-j \right|p(i,j)$$

3.9. Autocorrelation

$$autocorrelation=\sum_{i=1}^{N_{g}} \sum_{j=1}^{N_{g}} i\times j\times p(i,j)$$

3.10. Difference Entropy

$$difference entropy=-\sum_{i=0}^{N_{g}-1} \log_{2} {[p}_{x-y}(i)]\times p_{x-y}(i)$$

3.11. Sum Entropy

$$sum entropy=-\sum_{i=2}^{2N_{g}} \log_{2} {[p}_{x+y}(i)]\times p_{x+y}(i)$$

3.12. Sum Variance (where SE is sum entropy)

$$sum variance=\sum_{i=2}^{{2N}_{g}} {(i-SE)}^{2} p_{x+y}(i)$$

3.13. Cluster Prominence

$$cluster prominence=\sum_{i=1}^{N_{g}} \sum_{j=1}^{N_{g}} \left( i+j-\mu_{x}-\mu_{y} \right)^{4}p(i,j)$$

3.14. Cluster Shade

$$cluster shade=\sum_{i=1}^{N_{g}} \sum_{j=1}^{N_{g}} \left( i+j-\mu_{x}-\mu_{y} \right)^{3}p(i,j)$$

3.15. Cluster Tendency

$$cluster tendency=\sum_{i=1}^{N_{g}} \sum_{j=1}^{N_{g}} \left( i+j-\mu_{x}-\mu_{y} \right)^{2}p(i,j)$$

**4. Gray-level run-length matrix (GLRLM) based texture features**

A GLRLM of size N_g_ (=64) x N_r_ is denoted with ***P*** , where N_r_ is the number of different run lengths, and describes textural information of given ROI. The **P**(*i*, *j*) is defined as the number of runs with *j* consecutive voxels of intensity *i* with direction of δ. The distance vector δ is from $\left\{ \left( x,y,z \right)|x\in\left( -1,0,1 \right) and y\in\left( -1,0,1 \right) and z\in\left( -1,0,1 \right) and (x,y,z)\neq(0,0,0) \right\}$. In case of 2D features, δ is from $\left\{ \left( x,y \right)|x\in\left( -1,0,1 \right) and y\in\left( -1,0,1 \right) and (x,y)\neq(0,0) \right\}$. To account for discretization length differences, neighbors at a distance of 1, $\sqrt{2}$, $\sqrt{3}$ were given a weight of 1, $1/\sqrt{2}$, $1/\sqrt{3}$, respectively.

• p(i,j) = ${P(i,j)}/{\sum P(i,j)}$

• μ_x_ = mean of p_x_(i)

• μ_y_ = mean of p_y_(j)

• N = number of voxels

4.1. Short Run Emphasis

$$small run emphasis=\sum_{i=1}^{N_{g}} \sum_{j=1}^{N_{r}} \frac{p(i,j)}{j^{2}}$$

4.2. Long Run Emphasis

$$long run emphasis=\sum_{i=1}^{N_{g}} \sum_{j=1}^{N_{r}} j^{2}p(i,j)$$

4.3. Gray-Level Nonuniformity

$$gray level nonuniformity=\sum_{i=1}^{N_{g}} \left( \sum_{j=1}^{N_{r}} p(i,j) \right)^{2}$$

4.4. Run-Length Nonuniformity

$$run length nonuniformity=\sum_{j=1}^{N_{r}} \left( \sum_{i=1}^{N_{g}} p(i,j) \right)^{2}$$

4.5. Run Percentage

$$run percentage=\frac{1}{N}\sum_{i=1}^{N_{g}} \sum_{j=1}^{N_{r}} P(i,j)$$

4.6. Low Gray-Level Run Emphasis

$$low gray level run emphasis=\sum_{i=1}^{N_{g}} \sum_{j=1}^{N_{r}} \frac{p(i,j)}{i^{2}}$$

4.7. High Gray-Level Run Emphasis

$$high gray level run emphasis=\sum_{i=1}^{N_{g}} \sum_{j=1}^{N_{r}} i^{2}p(i,j)$$

4.8. Short Run Low Gray-Level Emphasis

$$short run low gray level emphasis=\sum_{i=1}^{N_{g}} \sum_{j=1}^{N_{r}} \frac{p(i,j)}{i^{2}j^{2}}$$

4.9. Short Run High Gray Level Emphasis

$$short run high gray level emphasis=\sum_{i=1}^{N_{g}} \sum_{j=1}^{N_{r}} \frac{i^{2}p(i,j)}{j^{2}}$$

4.10. Long Run Low Gray-Level Emphasis

$$long run low gray level emphasis=\sum_{i=1}^{N_{g}} \sum_{j=1}^{N_{r}} i^{2}j^{2}p(i,j)$$

4.11. Long Run High Gray-Level Emphasis

$$long run high gray level emphasis=\sum_{i=1}^{N_{g}} \sum_{j=1}^{N_{r}} \frac{j^{2}p(i,j)}{i^{2}}$$

4.12. Gray-Level Variance

$$gray level variane=\sum_{i=1}^{N_{g}} \sum_{j=1}^{N_{r}} {(i-\mu_{x})}^{2}p(i,j)$$

4.13. Run-Length Variance

$$run length variane=\sum_{i=1}^{N_{g}} \sum_{j=1}^{N_{r}} {(j-\mu_{y})}^{2}p(i,j)$$

**5. Gray-level size-zone matrix (GLSZM) based texture features**

A GLSZM of size N_g_ (=64) x N_z_ is denoted with ***P***, where N_z_ is the number of different zone sizes, and describes textural information of given ROI. A gray level zone is defined as the number of connected voxels with the same intensity. Voxels are considered connected if the distance between the two voxels is δ. The **P**(*i*, *j*) is defined as the number of zones with size of *j* and intensity of *i*. The distance vector δ is from $\left\{ \left( x,y,z \right)|x\in\left( -1,0,1 \right) and y\in\left( -1,0,1 \right) and z\in\left( -1,0,1 \right) and (x,y,z)\neq(0,0,0) \right\}$. In case of 2D features, δ is from $\left\{ \left( x,y \right)|x\in\left( -1,0,1 \right) and y\in\left( -1,0,1 \right) and (x,y)\neq(0,0) \right\}$. To account for discretization length differences, neighbors at a distance of 1, $\sqrt{2}$, $\sqrt{3}$ were given a weight of 1, $1/\sqrt{2}$, $1/\sqrt{3}$, respectively.

• p(i,j) = ${P(i,j)}/{\sum P(i,j)}$

• μ_x_ = mean of p_x_(i)

• μ_y_ = mean of p_y_(j)

• N = number of voxels

5.1. Small Zone Emphasis

$$small zone emphasis=\sum_{i=1}^{N_{g}} \sum_{j=1}^{N_{z}} \frac{p(i,j)}{j^{2}}$$

5.2. Large Zone Emphasis

$$large zone emphasis=\sum_{i=1}^{N_{g}} \sum_{j=1}^{N_{z}} j^{2}p(i,j)$$

5.3. Gray-Level Nonuniformity

$$gray level nonuniformity=\sum_{i=1}^{N_{g}} \left( \sum_{j=1}^{N_{z}} p(i,j) \right)^{2}$$

5.4. Zone-Size Nonuniformity

$$zone size nonuniformity=\sum_{j=1}^{N_{z}} \left( \sum_{i=1}^{N_{g}} p(i,j) \right)^{2}$$

5.5. Zone Percentage

$$zone percentage=\frac{1}{N}\sum_{i=1}^{N_{g}} \sum_{j=1}^{N_{z}} P(i,j)$$

5.6. Low Gray-Level Zone Emphasis

$$low gray level zone emphasis=\sum_{i=1}^{N_{g}} \sum_{j=1}^{N_{z}} \frac{p(i,j)}{i^{2}}$$

5.7. High Gray-Level Zone Emphasis

$$high gray level zone emphasis=\sum_{i=1}^{N_{g}} \sum_{j=1}^{N_{z}} i^{2}p(i,j)$$

5.8. Small Zone Low Gray-Level Emphasis

$$small zone low gray level emphasis=\sum_{i=1}^{N_{g}} \sum_{j=1}^{N_{z}} \frac{p(i,j)}{i^{2}j^{2}}$$

5.9. Small Zone High Gray-Level Emphasis

$$small zone high gray level emphasis=\sum_{i=1}^{N_{g}} \sum_{j=1}^{N_{z}} \frac{i^{2}p(i,j)}{j^{2}}$$

5.10. Large Zone Low Gray-Level Emphasis

$$large zone low gray level emphasis=\sum_{i=1}^{N_{g}} \sum_{j=1}^{N_{z}} i^{2}j^{2}p(i,j)$$

5.11. Large Zone High Gray-Level Emphasis

$$large zone high gray level emphasis=\sum_{i=1}^{N_{g}} \sum_{j=1}^{N_{z}} \frac{j^{2}p(i,j)}{i^{2}}$$

5.12. Gray-Level Variance

$$gray level variane=\sum_{i=1}^{N_{g}} \sum_{j=1}^{N_{z}} {(i-\mu_{x})}^{2}p(i,j)$$

5.13. Zone-Size Variance

$$zone size variane=\sum_{i=1}^{N_{g}} \sum_{j=1}^{N_{z}} {(j-\mu_{y})}^{2}p(i,j)$$

**6. Neighborhood gray-tone difference matrix (NGTDM) based texture features**

A NGTDM of size N_g_ (=64) x 1 is denoted with ***P***. The P(i) is defined as $\sum_{all voxels\in\{N_{i}\}} \left| i-\bar{A_{i}} \right|$ if $N_{i}>0$ and 0 if $N_{i}=0$, where {N_i_} is the set of all voxels with gray-level *i*, N_i_ is the number of voxels with gray-level *i*, and $\bar{A_{i}}$ is the average gray-level of the connected neighbors around voxels with intensity *i*. Voxels are considered connected if the distance between the two voxels is δ. The **P**(*i*, *j*) is defined as the number of zones with size of *j* and intensity of *i*. The distance vector δ is from $\left\{ \left( x,y,z \right)|x\in\left( -1,0,1 \right) and y\in\left( -1,0,1 \right) and z\in\left( -1,0,1 \right) and (x,y,z)\neq(0,0,0) \right\}$. In case of 2D features, δ is from $\left\{ \left( x,y \right)|x\in\left( -1,0,1 \right) and y\in\left( -1,0,1 \right) and (x,y)\neq(0,0) \right\}$. To account for discretization length differences, neighbors at a distance of 1, $\sqrt{2}$, $\sqrt{3}$ were given a weight of 1, $1/\sqrt{2}$, $1/\sqrt{3}$, respectively.

• N = number of voxels

• N_e_ = effective number of gray-levels

• $\epsilon$ = small number to prevent infinite

6.1. Coarseness

$$coarseness=\frac{N}{\epsilon+\sum_{i=1}^{N_{g}} N_{i}P(i)}$$

6.2. Contrast

$$contrast=\left[ \frac{1}{N^{3}}\sum_{i=1}^{N_{g}} P(i) \right]\left[ \frac{1}{N_{e}(N_{e}-1)}\sum_{i=1}^{N_{g}} \sum_{j=1}^{N_{g}} N_{i}N_{j}{(i-j)}^{2} \right]$$

6.3. Busyness

$$busyness=\frac{\sum_{i=1}^{N_{g}} N_{i}P(i)}{\sum_{i=1}^{N_{g}} \sum_{j=1}^{N_{g}} (iN_{i}-jN_{j})}, N_{i}\neq0, N_{j}\neq0$$

6.4. Complexity

$$complexity=\sum_{i=1}^{N_{g}} \sum_{j=1}^{N_{g}} \frac{\left| i-j \right|\left[ N_{i}P\left( i \right)+N_{j}P(j) \right]}{N(N_{i}+N_{j})}, N_{i}\neq0, N_{j}\neq0$$

6.5. Strength

$$strength=\frac{\sum_{i=1}^{N_{g}} \sum_{j=1}^{N_{g}} (N_{i}+N_{j}){(i-j)}^{2}}{N\times\left[ \epsilon+\sum_{i=1}^{N_{g}} P(i) \right]}, N_{i}\neq0, N_{j}\neq0$$

**Table S1: The 55 extracted features from region of interest**

| Type | Features | Number |
| --- | --- | --- |
| Geometry | Volume | 1 |
| First-order features | Variance, Skewness, Kurtosis, Energy, Entropy, Interquartile range, Mean, Uniformity | 8 |
| Gray-level co-occurrence matrix texture features | Energy, Contrast, Entropy, Homogeneity, Correlation, Sum average, Variance, Dissimilarity, Auto-correlation, Difference entropy, Sum entropy, Sum variance, Cluster prominence, Cluster shade, Cluster tendency | 15 |
| Gray-level run-length matrix texture features | Short run emphasis, Long run emphasis, Gray-level nonuniformity, Run-length nonuniformity, Run percentage, Low gray-level run emphasis, High gray-level run emphasis, Short run low gray-level emphasis, Short run high gray-level emphasis, Long run low gray-level emphasis, Long run high gray-level emphasis, Gray-level variance, Run-length variance | 13 |
| Gray-level size-zone matrix texture features | Small zone emphasis, Large zone emphasis, Gray-level nonuniformity, Zone-size nonuniformity, Zone percentage, Low gray-level zone emphasis, High gray-level zone emphasis, Small zone low gray-level emphasis, Small zone high gray-level emphasis, Large zone low gray-level emphasis, Large zone high gray-level emphasis, Gray level variance, Zone-size variance | 13 |
| Neighbor gray-tone difference matrix texture features | Coarseness, Contrast, Busyness, Complexity, Strength | 5 |

**Table S2: Selected features from robustness test**

| Type | Features | Number |
| --- | --- | --- |
| Geometry | Volume | 1 |
| First-order features | Energy, Mean | 2 |
| Gray-level co-occurrence matrix texture features | Energy, Homogeneity, Sum average, Auto-correlation, Sum variance, Cluster tendency | 6 |
| Gray-level run-length matrix texture features | Long run emphasis, Low gray-level run emphasis, High gray-level run emphasis, Short run low gray-level emphasis, Short run high gray-level emphasis, Long run low gray-level emphasis, Long run high gray-level emphasis | 7 |
| Gray-level size-zone matrix texture features | Large zone emphasis, Low gray-level zone emphasis, Small zone low gray-level emphasis, Large zone low gray-level emphasis, Large zone high gray-level emphasis | 5 |
| Neighbor gray-tone difference matrix texture features | Coarseness | 1 |

**Table S3: Performance of the radiomics signatures in training and validation cohorts**

| Radiomics signature | Cohort | AUC | Hosmer-Lemeshow p-value |
| --- | --- | --- | --- |
| LR 3D Radscore | Training | 0.901 | 0.59 |
|  | Validation | 0.937 | 0.34 |
| LR 2D Radscore | Training | 0.835 | 0.45 |
|  | Validation | 0.843 | 0.99 |
| DM 3D Radscore | Training | 0.737 | 0.99 |
|  | Validation | 0.747 | 0.99 |
| DM 2D Radscore | Training | 0.761 | 0.16 |
|  | Validation | 0.801 | 0.66 |
| DFS 3D Radscore | Training | 0.732 | 0.94 |
|  | Validation | 0.744 | 0.27 |
| DFS 2D Radscore | Training | 0.756 | 0.18 |
|  | Validation | 0.784 | 0.90 |

**Appendix Table D.1: Results of univariate and multivariate analyses for local recurrence**

|  |  | 3D |  | 2D |  |
| --- | --- | --- | --- | --- | --- |
|  | Univariate | Multivariate |  | Multivariate |  |
| Characteristics | P-value | HR (95% CI) | P-value | HR (95% CI) | P-value |
| Age | 0.84 | - | - | - | - |
| Sex | 0.27 | - | - | - | - |
| cT stage (T1-3 vs. T4) | 0.03 | 1.19 (0.11-12.67) | 0.88 | 1.24 (0.18-8.59) | 0.83 |
| cN stage (N0 vs. N1-2) | 1 | - | - | - | - |
| CEA | 0.08 | - | - | - | - |
| ypT stage (T0-2 vs. T3-4) | 0.04 | 5.06 (0.52-49.58) | 0.16 | 3.34 (0.48-23.35) | 0.22 |
| ypN stage (N0 vs. N1-2) | 0.01 | 3.11 (0.61-15.92) | 0.17 | 1.24 (0.25-6.29) | 0.79 |
| CRM (<1mm) | 0.5 | - | - | - | - |
| Lymphatic invasion | 0.004 | 6.41 (0.29-140.26) | 0.24 | 1.11 (0.12-10.26) | 0.93 |
| Venous invasion | 0.008 | 0.84 (0.07-9.77) | 0.89 | 3.23 (0.44-23.61) | 0.25 |
| Perineural invasion | 0.03 | 0.40 (0.04-4.28) | 0.45 | 1.25 (0.21-7.33) | 0.81 |
| Dworak TRG (1-2 vs. 3-4) | 0.09 | - | - | - | - |
| 3D Rad-score | 0.00001 | 4.34 (1.83-10.28) | 0.0008* | - | - |
| 2D Rad-score | 0.00005 | - | - | 9.17 (2.45-34.31) | 0.001* |

**Appendix Table D.2: Results of univariate and multivariate analyses for distant metastasis**

|  |  | 3D |  | 2D |  |
| --- | --- | --- | --- | --- | --- |
|  | Univariate | Multivariate |  | Multivariate |  |
| Characteristics | P-value | HR (95% CI) | P-value | HR (95% CI) | P-value |
| Age | 0.34 | - | - | - | - |
| Sex | 0.92 | - | - | - | - |
| cT stage (T1-3 vs. T4) | 0.06 | - | - | - | - |
| cN stage (N0 vs. N1-2) | 0.19 | - | - | - | - |
| CEA | 0.03 | 2.12 (0.87-5.14) | 0.10 | 2.13 (0.88-5.19) | 0.10 |
| ypT stage (T0-2 vs. T3-4) | 0.009 | 0.82 (0.23-2.93) | 0.75 | 0.68 (0.20-2.37) | 0.55 |
| ypN stage (N0 vs. N1-2) | 0.01 | 1.95 (0.69-5.47) | 0.20 | 1.90 (0.70-5.15) | 0.21 |
| CRM (<1mm) | 0.42 | - | - | - | - |
| Lymphatic invasion | 0.006 | 8.15 (1.16-57.23) | 0.03* | 10.62 (1.60-70.28) | 0.01* |
| Venous invasion | 0.04 | 1.13 (0.32-4.03) | 0.85 | 1.60 (0.44-5.75) | 0.47 |
| Perineural invasion | 0.002 | 1.44 (0.49-4.22) | 0.50 | 1.44 (0.49-4.22) | 0.51 |
| Dworak TRG (1-2 vs. 3-4) | 0.01 | 0.27 (0.05-1.38) | 0.12 | 0.17 (0.03-0.87) | 0.03* |
| 3D Rad-score | 0.00007 | 4.61 (1.64-12.97) | 0.004* | - | - |
| 2D Rad-score | 0.002 | - | - | 3.09 (1.27-7.53) | 0.01* |

**Appendix Table D.3: Results of univariate and multivariate analyses for disease-free survival**

|  |  | 3D |  | 2D |  |
| --- | --- | --- | --- | --- | --- |
|  | Univariate | Multivariate |  | Multivariate |  |
| Characteristics | P-value | HR (95% CI) | P-value | HR (95% CI) | P-value |
| Age | 0.31 | - | - | - | - |
| Sex | 0.95 | - | - | - | - |
| cT stage (T1-3 vs. T4) | 0.01 | 2.69 (0.81-8.88) | 0.10 | 2.11 (0.65-6.85) | 0.21 |
| cN stage (N0 vs. N1-2) | 0.18 | - | - | - | - |
| CEA | 0.02 | 2.22 (0.93-5.31) | 0.07 | 2.19 (0.91-5.25) | 0.08 |
| ypT stage (T0-2 vs. T3-4) | 0.006 | 0.76 (0.21-2.70) | 0.67 | 0.64 (0.19-2.21) | 0.48 |
| ypN stage (N0 vs. N1-2) | 0.006 | 1.88 (0.67-5.25) | 0.23 | 1.82 (0.67-4.98) | 0.24 |
| CRM (<1mm) | 0.47 | - | - | - | - |
| Lymphatic invasion | 0.007 | 5.93 (0.78-45.05) | 0.09 | 9.22 (1.37-61.92) | 0.02* |
| Venous invasion | 0.05 | 0.79 (0.20-3.13) | 0.73 | 1.15 (0.30-4.43) | 0.84 |
| Perineural invasion | 0.002 | 1.50 (0.51-4.41) | 0.46 | 1.57 (0.54-4.60) | 0.41 |
| Dworak TRG (1-2 vs. 3-4) | 0.008 | 0.22 (0.05-1.07) | 0.06 | 0.16 (0.03-0.78) | 0.02* |
| 3D Rad-score | 0.0001 | 6.62 (1.98-22.20) | 0.002* | - | - |
| 2D Rad-score | 0.002 | - | - | 3.25 (1.29-8.19) | 0.01* |
